# The Economic Burden of Self-Reported and Undiagnosed Cardiovascular Diseases and Diabetes on Indonesian Households

**DOI:** 10.1371/journal.pone.0099572

**Published:** 2014-06-10

**Authors:** Eric A. Finkelstein, Junxing Chay, Shailendra Bajpai

**Affiliations:** 1 Health Services and Systems Research Program, Duke-NUS Graduate Medical School, Singapore, Singapore; 2 Global Health Institute, Duke University, Durham, North Carolina, United States of America; 3 Medical Affairs, Sanofi-aventis, Singapore, Singapore; Monash University, Australia

## Abstract

**Objectives:**

The goal of this study is: (1) to estimate the current direct out-of-pocket (OOP) and indirect non-communicable diseases (NCD) burden on Indonesian households and (2) to project NCD prevalence and burden in 2020 focusing specifically on hypertension, diabetes, heart problems and stroke.

**Methods:**

This study relies on econometric analyses based on four waves of the Indonesian Family Life Survey (IFLS).

**Results:**

In aggregate, of the NCDs studied, heart problems exert the greatest economic burden on households, costing Int$1.56 billion in OOP and indirect burden in 2010. This was followed by hypertension (Int$1.36 billion), diabetes (Int$0.81 billion) and stroke (Int$0.29 billion). The OOP and indirect burden of these conditions is estimated to be Int$4.02 billion. Diabetes and stroke are expected to have the largest proportional increases in burden by 2020; 56.0% for diabetes and 56.9% for stroke to total Int$1.27 billion and Int$0.45 billion respectively. The burden of heart problems in 2020 is expected to increase by 34.4% to total Int$2.09 billion and hypertension burden will increase by 46.1% to Int$1.99 billion. In 2020, these conditions are expected to impose an economic burden of Int$5.80 billion.

**Conclusion:**

In conclusion, this study demonstrates the significant burden of 4 primary NCDs on Indonesian households. In addition to the indirect burden, hypertension, diabetes, heart problems and stroke account for 8% of the nation's OOP healthcare expenditure, and due to rising disease prevalence and an aging population, this figure is expected to increase to 12% by 2020 without a significant health intervention.

## Introduction

In a span of only a few decades, the burden of disease in low and middle income countries (LMICs) has transformed from primarily infectious diseases and injuries to non-communicable diseases (NCDs). Today, almost 80% of NCD deaths worldwide occur in low and middle-income countries, with the leading cause being cardiovascular disease [1]. In South-East Asia specifically, the prevalence of NCDs and NCD-related mortality is anticipated to increases by more than 20% over the next decade [1]. In Indonesia, where smoking rates are among the highest in the region, the burden of NCDs is likely to be even larger.

Understanding the health and economic implications of the growing NCD epidemic is critical for resource planning. Yet, although many burden-of-disease studies on NCDs have been published for developed countries [2]–[12] there are relatively few in developing countries [13]–[17] and no nationally representative studies focusing on NCDs in Indonesia. We identified only two potentially relevant studies. Both focused solely on the medical costs attributed to smoking [18]–[19]. This is a significant shortcoming as Indonesia, a country with over 242 million residents, is currently undergoing major healthcare reforms and estimates of current and future NCD burden will prove useful in predicting future public and private sector health expenditures for NCDs and for identifying the potential savings from successful public health interventions aimed at reducing NCD incidence.

To address this shortcoming, this study has 2 objectives: (1) to estimate the current direct out-of-pocket (OOP) and indirect NCD burden on Indonesian households and (2) to project NCD prevalence and associated burden in 2020. NCDs considered in this study include hypertension, diabetes, heart problems and stroke. The direct financial burden includes healthcare utilization and OOP expenditures. The indirect burden includes loss of productivity and required assistance in daily living. Unless clearly specified otherwise, the term “prevalence” in this study refers to the combined prevalence of self-reported and undiagnosed cases.

## Methods

### Data

The Indonesian Family Life Survey (IFLS) is an ongoing health and socioeconomic longitudinal survey consisting of 4 waves, the first one beginning in 1993. The original sampling frame was based on households in 13 of the 26 Indonesian provinces, representing about 83% of the Indonesian population. IFLS-1 collected information from over 22,000 individuals living in 7,224 households. These original households and their split-offs were followed in subsequent waves fielded in 1997/98, 2000 and 2007/08. By the fourth wave, the survey included 44,103 individuals from 13,535 households.

Each wave included individual-level data on anthropometric measurements, morbidity indicators, healthcare utilization, health behaviors, employment, and household income (comprising net profits from farm and non-farm businesses, and wages received). The survey also involved a physical examination performed by a qualified health worker. Because NCD-related questions were only posed to individuals aged 40 and over (n = 10,795 in IFLS-4), this analysis is limited to that sample.

Input variables used in the estimation include demographics (age, gender, ethnicity, educational attainment, employment status, and annual personal and household income); self-reports of NCDs and risk factors (conditions symptomatic of diabetes and angina, current tobacco use and time spent on physical activities of vigorous and moderate intensities); and measured variables from the physical examination (body mass index (BMI), blood pressure and cholesterol values). Outcome variables include frequency of outpatient and inpatient visits, OOP expenditures for medical treatments and medications (prescription and non-prescription); number of days of primary activity missed due to poor health, and the number of hours of assistance required in performing basic and instrumental activities in daily living (ADLs). The recall period for inpatient utilization was the past 12 months. For outpatient utilization, self-medication, number of days of primary activity missed, and number of hours of assistance in ADLs, the recall period was the past 4 weeks. All measures were annualized. Monetary values were adjusted for inflation and converted to 2012 International Dollars.

### Classification criteria

Respondents were classified as hypertensive if their blood pressure measurements met the JNC VII criteria of having a systolic blood pressure reading of 140 and above and/or a diastolic blood pressure reading of 90 and above [20]. Respondents were considered to have a high risk of obesity-related disease using the Asian BMI cutoff of 27.5 or greater [21]. High blood cholesterol is defined as having total cholesterol greater than 240 mg/dL and low high-density lipoprotein (HDL) cholesterol as less than 40 mg/dL. Respondents were classified as insufficiently physically active if they did not meet the World Health Organization (WHO) recommended level of physical activity: 150 min of moderate-intensity activity or 75 min of vigorous-intensity activity or any equivalent combinations of moderate- and vigorous-intensity activity throughout the week [22].

Among respondents with no reported diabetes or heart problems, undiagnosed diabetes and heart problems were identified based on the number of affirmative responses to conditions symptomatic of the respective disease. For diabetes, these are (a) often wake up to urinate during the night (b) a cut or wound takes a long time to heal (c) often have headache when waking up in the morning. For heart problems, these are (a) ever felt pain on the left side of your chest (b) ever felt chest pains when climbing up stairs or uphill (c) ever felt chest pains when being active or walking fast. Undiagnosed diabetes is defined as having all 3 symptoms; undiagnosed heart problem is defined as having at least 2 symptoms. No attempt was made to identify undiagnosed stroke patients as it is assumed that those who had a stroke will be aware of their condition.

### Statistical analyses

Current and future aggregate burden of NCDs in Indonesia was estimated by multiplying the per capita average burden by the projected NCD prevalence rate. The human capital approach was used for monetizing the indirect burden. The lost days of primary activity were valued at the average daily labour rate in Indonesia (Int$3.48), which includes self-employment income and income for those who work for pay. Time spent assisting activities of daily living was valued at the hourly market rate for paid assistance (Int$0.86), as is the common practice [23]–[25].

In order to quantify the incremental effect of each included NCDs, multivariate analyses were conducted to control for possible confounding. A negative binomial regression model was used for the frequency of healthcare utilization, number of days of primary activity missed and number of hours of assistance required for daily living activities. For OOP expenditures, a 2-step model consisting of a logistic regression in the first step and a generalized linear model with log-link and gamma-distributed error was used in the second step. This approach is commonly used to estimate incremental costs for select diseases and risk factors [4], [26]–[27]. All estimates controlled for the same set of confounders: age, gender, ethnicity, educational attainment and other self-reported NCDs. Since IFLS-4 was not representative of the entire Indonesian population, estimates were weighted based on prevalence rates (within age and gender strata) obtained from the 2007 census [28].

For each model, the burden due to NCDs was estimated as the mean marginal difference of the predicted outcome with the NCD dummy switched on or off. This allowed for estimating the ‘counterfactual’ of what the burden would have been in the absence of NCDs while leaving the other model parameters unchanged. For the negative binomial and logistic regression models, standard errors were obtained using the delta method and statistical significance determined by a Wald test. For the 2-step model, we used the non-parametric bootstrapping method (1,200 replications).

Projection of future NCD prevalence took into consideration prevailing trends within age and gender strata and changes in the future demographic profile. Because questions on NCDs were only introduced in IFLS-4, NCD prevalence in waves prior to IFLS-4 were predicted based on age, BMI and tobacco use. A multinomial logistic regression was then used to estimate the probabilities of having each NCD. Estimates for males and females were conducted separately to account for differences in trajectories. Age- and gender-specific time trends were then identified using a linear time-trend extrapolated to 2020. National NCD prevalence was then calculated by applying the projected prevalences within strata to population demographics in 2020 [28]. Confidence intervals for future NCD prevalence were obtained via bootstrapping (1,200 replications). The aggregate annual burden is calculated by multiplying the estimated per capita NCD burden by its projected prevalence. Confidence intervals around the aggregate burden estimates were calculated using Monte Carlo simulation methods, assuming independent normal distributions for NCD prevalence and per capita burden and bootstrap standard errors as the standard deviation.

All statistical analyses were performed using Stata (version MP 12.1).

### Ethics statement

This study is based on publicly available de-identified data. It was approved by the National University of Singapore Institutional Review Board.

## Results

### IFLS-4 sample characteristics

Table 1 presents demographic characteristics of respondents. The mean age among IFLS-4 respondents used in this study is 53.8 with 47.2% being male. There are about 300 distinct ethnic groups in Indonesia, with Javanese and Sundanese being the largest groups. The remaining ethnic groups individually make up less than 5% of the total population. 75.0% of IFLS-4 respondents were employed and earned an average annual income of Int$1,650.

**Table 1:** Sample demographics (n = 10,795).

Variables		Mean/percentage [95% CI]
Age (mean)		53.3 [53.1, 53.5]
Male (%)		47.8 [46.8, 48.7]
Ethnicity (%)		
	Javanese	44.8 [43.9, 45.8]
	Sundanese	12.1 [11.5, 12.7]
	Others	43.0 [42.1, 44.0]
Employed (%)		75.9 [75.1, 76.7]
Personal income among employed individuals ($Int)		1674 [1592, 1755]
Household income (Int$)		3179 [3122, 3254]
Educational attainment (%)		
	None	15.8 [15.2, 16.5]
	Elementary school	50.8 [49.9, 51.8]
	High school	25.3 [24.4, 26.1]
	College/university	7.5 [7.0, 8.0]
	Others	0.6 [0.4, 0.7]

Table 2 presents prevalence rates for NCDs and risk factors for this sample. The prevalence of heart problems is 14.6%, diabetes prevalence is 4.5% and stroke prevalence is 1.0%. Concerning NCD risk factors, hypertension has the highest prevalence at 52.7%. Obesity prevalence is 14.6%, 15.7% have high blood cholesterol and 54.7% have low HDL levels. Smokers make up 35.2% of the sample and 40.1% are not sufficiently active. Our estimates for undiagnosed CVD and diabetes match those from the National Basic Health Research survey (Riskesdas) [29], lending some support to the validity our classification method for undiagnosed cases.

**Table 2:** Sample prevalence of risk factors and NCDs from IFLS and Riskesdas.

			IFLS 2007/08 (n = 10,795)	Riskesdas§ 2007 (n = 987,205)
Variables			Prevalence [95% CI]	Prevalence
Risk factors associated with metabolic syndrome				
	Obesity (%)		14.7 [14.0, 15.4]	23.2
	Cholesterol level			
		High blood cholesterol (%)	15.6 [14.9, 16.3]	
		Low HDL (%)	55.0 [54.0, 55.9]	
	Tobacco consumption habit (%)		35.5 [34.6, 36.4]	36.9
	Insufficient physical activity (%)		39.4 [38.5, 40.4]	46.6
Non-communicable disease				
	Hypertension (%)			
		Self-reported	18.4 [17.6, 19.1]	16.1
		Undiagnosed	33.8 [32.9, 34.7]	35.1
	Diabetes (%)			
		Self-reported	2.9 [2.6, 3.3]	2.3
		Undiagnosed	1.6 [1.4, 1.9]	0.8
	Heart problems (%)			
		Self-reported	2.3 [2.0, 2.6]	2.4
		Undiagnosed	12.2 [11.6, 12.8]	12.9
	Stroke (%)			
		Self-reported	0.9 [0.7, 1.1]	1.5

§ Household were sampled from the entire Republic of Indonesia.

### Per capita burden

Table 3 presents results of the regression analyses. In most cases, self-reported NCDs have a positive and statistically significant effect on outpatient and inpatient utilization and OOP costs. Diabetes imposes the largest burden on outpatient expenditures of Int$67 (95% CI 15 to 45) per year. Stroke imposes the greatest burden for inpatient expenditures (Int$42per year, 95% CI 1 to 140). The burden on the number of days of primary activity missed and the number of hours of assistance required for ADLs were significantly positive among all the self-reported NCDs. Hypertension imposed the smallest burden while stroke imposed the greatest burden.

**Table 3:** Annual per capita NCD burden in 2007/08 by type of disease and disease awareness.

Annual predicted burden			Hypertension		Diabetes		Heart problems		Stroke
			SR [95% CI]	UD [95% CI]	SR [95% CI]	UD [95% CI]	SR [95% CI]	UD [95% CI]	SR [95% CI]
Direct									
	Frequency of healthcare utilization								
		Outpatient	1.9 [1.2, 2.6] *	−0.6 [−1.1, −0.1]*	4.1 [1.6, 6.5] *	−0.7 [−1.8, 0.4]	3.5 [1.5, 5.5] *	1.9 [0.9, 2.8] *	2.4 [0.2, 4.6] *
		Inpatient	0.0 [0.0, 0.0]	0.0 [0.0, 0.0]	0.1 [0.0, 0.1] *	0.0 [0.0, 0.0]	0.1 [0.0, 0.1] *	0.0 [0.0, 0.0]	0.1 [0.0, 0.1] *
	Expected OOP medical expense (Int$)								
		Outpatient	14 [6], [23] *	1 [−5, 7]	67 [15, 145] *	−9 [−15, −1] *	28 [8, 57] *	8 [−1, 17]	22 [−1, 53]
		Inpatient	10 [−1, 21]	20 [6], [37] *	22 [1, 61] *	−12 [−22, 5]	4 [−11, 28]	14 [−2, 36]	42 [1, 140] *
		Self-treatment	3 [−1, 8]	−1 [−5, 4]	17 [5], [33] *	4 [−1, 10]	3 [−1, 10]	5 [2], [8] *	5 [−3, 19]
Indirect									
	Number of days of primary daily activity		8 [5], [12] *	−2 [−5, 1]	26 [14], [38] *	10 [1], [19] *	21 [11, 91] *	18 [14], [23] *	35 [13, 56] *
	Number of hours of assistance required		38 [3, 74] *	17 [−14, 47]	108 [20, 197] *	39 [−63, 142]	128 [21, 234] *	80 [35, 125] *	260 [73, 447] *
Total direct † (Int$)			30 [10, 50] *	10 [−10, 20]	130 [60, 230] *	−10 [−30, 0]	50 [10, 100] *	20 [0, 40] *	80 [20, 160] *
Total indirect § (Int$)			50 [20, 70] *	0 [−20, 30]	160 [90, 220] *	50 [−10, 120]	170 [100, 260] *	110 [80, 140] *	280 [160, 410] *
Combined burden ‡ (Int$)			70 [40, 100] *	10 [−20, 50]	330 [190, 520] *	40 [−20, 110]	190 [110, 290] *	130 [100, 180] *	380 [230, 560] *
Combined burden # (%)			2.1	0.3	5.9	1.8	4.7	5.5	10.7

SR Self-reported; UD Undiagnosed.

Two-tailed significance test from zero were performed. P-values below 5% is indicated with an asterisk.

† Sum of out-of-pocket expenditures for outpatient, inpatient and self-treatment rounded to the nearest Int$10.

§ Sum of monetized indirect burden, rounded to the nearest Int$10. Each day of lost primary activity was valued at the average expected daily wage (Int$3.86) and each of hour of required assistance valued at the hourly market rate for paid assistance (Int$0.34).

‡ Sum of direct and indirect burdens, rounded to the nearest Int$10.

# A percentage of mean annual household income among each NCD status.

The burden of undiagnosed NCDs on healthcare utilization and costs was generally small and not statistically significant. This results because these cases are expected to be less severe, less likely to be treated, and/or most likely to suffer from measurement error. Even with these caveats, undiagnosed diabetes and heart problems significantly reduced primary activity by 10 (95% CI 1 to 19) and 18 (95% CI 14 to 23) days per year respectively.

All 4 self-reported NCDs demonstrated a statistically significant and positive per capita direct OOP and indirect burden. Hypertension had the smallest combined annual burden (Int$70, 95% CI 40 to 100), followed by heart problems (Int$190, 95% CI 110 to 290), diabetes (Int$330, 95% CI 190 to 520) and stroke (Int$380, 95% CI 230 to 560). Despite having similar values, the composition of the total burden of self-reported diabetes and stroke differed. Less than half of the combined burden for stroke comes from the direct OOP financial burden whereas the direct OOP and indirect burden for diabetes are close in value. Among undiagnosed NCDs only heart problems had a significant combined burden. Undiagnosed diabetes imposed a modest indirect burden (Int$50, 95% CI −10 to 120]), although we could not reject the hypothesis of no burden.

The combined burdens were also expressed as a percentage of the mean household income of those who have the disease. Stroke was the most burdensome to households, costing 10.7% of household income. This was followed by self-reported diabetes (5.9%) and heart problems (4.7%). The most costly undiagnosed NCD was heart problems (5.5%). Undiagnosed hypertension and diabetes do not appear to be particularly burdensome in terms of household income, costing 0.3% and 1.8% respectively.

### Trends of NCD prevalence by age and gender stratum

Estimates of gender- and age-specific linear time trends for self-reported and undiagnosed NCDs over the period 1994–2008 were obtained via least squares. Prevalences of self-reported NCDs were increasing over time for each age and gender stratum, and this upward trend is generally significant across the strata. Women were found to have consistently higher self-reported hypertension than men, with mostly higher baseline prevalence rates and annual increases across age groups. Yet the undiagnosed hypertension rates for men were higher at baseline for ages 40–64, and have increased at a faster rate than women across age groups. Self-reported diabetes experienced the greatest increase, relative to baseline, among individuals aged 40–74. Heart problems had the highest ratio of undiagnosed to self-reported cases and are consistently high across age and gender strata. Undiagnosed cases of heart problems were found to be decreasing with time, perhaps due to improved access to care. The assumption of a linear time trend appears reasonable given the information presented and the relatively short time horizon considered. The regression equations used to forecast future prevalence is reported in Table S1.

### National prevalence and economic burden

Table 4 combines the per capita cost estimates with future prevalence rates to generate NCD OOP and indirect burden in 2020 among those aged 40 and over. The Indonesian population above age 40 is projected to increase by 34.4% from 73.4 to 98.7 million in 2020, whereas total population growth is expected to be only 9.7%. Between 2010 and 2020 total hypertension prevalence is expected to increase by 6.8% (3.7 percentage points) as a result of similar increases in self-reported and undiagnosed cases. Prevalence of stroke is expected to increase by 20% (0.2 percentage points). Diabetes prevalence is expected to increase by 10.2% (0.5 percentage points) due to larger increases in self-reported cases. Self-reported heart problems is predicted to increase by 8% (0.2 percentage points).

**Table 4:** National NCD prevalence and burden in 2010 and 2020 among those aged 40+.

NCD		Prevalence (%) [95% CI]		Annual economic burden (Int$ in billions) [95% CI]					
		2010	2020	2010			2020		
				Direct	Indirect	Combined	Direct	Indirect	Combined
Hypertension									
	Self-reported	19.3 [18.6, 20.1]	21.3 [20.5, 22.0]	0.38 [0.14, 63]	0.68 [0.34, 1.02]	1.01 [0.53, 1.50]	0.57 [0.21, 0.93]	1.00 [0.50, 1.51]	1.50 [0.79, 2.21]
	Undiagnosed	34.9 [34.0, 35.8]	36.6 [35.8, 37.5]	0.15 [−0.21, 0.51]	0.11 [−0.43, 0.64]	0.35 [−0.49, 1.18]	0.21 [−0.30, 0.73]	0.15 [−0.60, 0.91]	0.49 [−0.69, 1.67]
	Total	54.2 [53.3, 55.2]	57.9 [57.0, 58.8]	0.54 [0.10, 0.97]	0.78 [0.15, 1.42]	1.36 [0.39, 2.32]	0.78 [0.15, 1.41]	1.15 [0.24, 2.06]	1.99 [0.61, 3.36]
Diabetes									
	Self-reported	3.2 [2.9, 3.5]	3.8 [3.4, 4.1]	0.31 [0.11, 0.52]	0.37 [0.21, 0.53]	0.77 [0.38, 1.17]	0.49 [0.17, 0.82]	0.58 [0.34, 0.84]	1.21 [0.60, 1.83]
	Undiagnosed	1.7 [1.4, 1.9]	1.6 [1.4, 1.9]	−0.02 [−0.03, 0.00]	0.06 [−0.01, 0.14]	0.04 [−0.04, 0.12]	−0.02 [−0.04, 0.00]	0.08 [−0.02, 0.19]	0.06 [−0.05, 0.17]
	Total	4.9 [4.5, 5.3]	5.4 [5.0, 5.8]	0.29 [0.09, 0.50]	0.43 [0.26, 0.61]	0.81 [0.42, 1.22]	0.47 [0.14, 0.80]	0.67 [0.40, 0.94]	1.27 [0.65, 1.90]
Heartproblems									
	Self-reported	2.4 [2.1, 2.7]	2.6 [2.3, 2.9]	0.08 [0.01, 0.16]	0.30 [0.16, 0.45]	0.34 [0.18, 0.51]	0.12 [0.01, 0.24]	0.44 [0.24, 0.66]	0.50 [0.27, 0.75]
	Undiagnosed	12.4 [11.7, 13.0]	12.0 [11.4, 12.7]	0.18 [0.01, 0.36]	1.00 [0.73, 1.27]	1.22 [0.85, 1.59]	0.24 [0.02, 0.47]	1.30 [0.96, 1.66]	1.59 [1.12, 2.07]
	Total	14.8 [14.1, 15.4]	14.7 [14.0, 15.4]	0.27 [0.08, 0.46]	1.30 [1.00, 1.60]	1.56 [1.16, 1.96]	0.36 [0.11, 0.62]	1.75 [1.34, 2.16]	2.09 [1.56, 2.63]
Stroke									
	Self-reported	1.0 [0.7, 1.3]	1.2 [0.9, 1.5]	0.06 [0.01, 0.12]	0.21 [0.10, 0.33]	0.29 [0.14, 0.45]	0.09 [0.01, 0.18]	0.32 [0.16, 0.52]	0.45 [0.23, 0.70]
Grand total				1.16 [0.63, 1.68]	2.72 [1.99, 3.46]	4.02 [2.89, 5.15]	1.71 [0.95, 2.46]	3.89 [2.84, 4.94]	5.80 [4.17, 7.42]

In aggregate, of the NCDs studied, heart problems exert the greatest economic burden on society, costing Int$1.56 billion (CI 1.16 to 1.96) in OOP and indirect burden in 2010. This was followed by hypertension (Int$1.36 billion, CI 0.39 to 2.32), diabetes (Int$0.81 billion, CI 0.42 to 1.22) and stroke (Int$0.29 billion, CI 0.14 to 0.45). The OOP and indirect burden of these conditions is estimated to be Int$4.02 billion (CI 2.89 to 5.15). Diabetes and stroke are expected to have the largest proportional increases in burden by 2020; 56.0% for diabetes and 56.9% for stroke to total Int$1.27 billion (CI 0.65 to 1.90) and Int$0.45 billion (CI 0.23 to 0.70) respectively. The burden of heart problems in 2020 is expected to increase at the same rate of population growth: 34.4% to total of Int$2.09 billion (CI 1.56 to 2.63). Hypertension burden in 2020 will increase by 46.1% to Int$1.99 billion (CI 0.61 to 3.36). In 2020, these conditions are expected to impose an economic burden of Int$5.80 billion (CI 4.17 to 7.42).

## Discussion

The goal of this study was to estimate the current direct OOP and indirect NCD burden in Indonesia and to project NCD prevalence and associated burden in 2020. We focused on 4 common NCDs: hypertension, diabetes, heart problems and stroke. We found that these NCDs exert considerable burdens on households, with estimates ranging between 4.7% (heart problems) and 10.7% (stroke) of household incomes among those who are aware of their conditions. In aggregate, OOP and indirect costs for these conditions in 2010 were estimated to be Int$4.02 billion and that this estimate is anticipated to increase to Int$5.80 billion (44% increase) over the next decade due to changing demographics and a rising disease incidence. According to WHO [30], Indonesia spent Int$29.5 billion in 2010 on health care expenditures. Of this total, Int$10.6 billion (36%) was spent by the government while Int$14.2 billion (48%) were OOP expenditures by individuals. The estimated direct OOP burden of these 4 NCDs made up approximately 8% (Int$1.2 billion) of the nation's OOP healthcare expenditure, and would increase to 12% (Int$1.7 billion) in 2020 based on our forecasts.

The International Diabetes Federation (IDF) projected diagnosed and undiagnosed diabetes prevalence across all ages to be 4.6% in 2010 and to rise to 6.0% in 2030 [31]. Our estimates for 2010 and 2020 were 4.9% and 5.4% respectively, in line with their estimates. They also predicted total (not OOP) health expenditures associated with diabetes to increase by roughly 75%. We predict OOP expenditures to increase by 62%. Because their estimate includes expenditures on public programs and health research, in addition to total direct expenditures, and ours focuses solely on OOP expenditures, the difference in results is not unexpected.

In addition to prediction total OOP expenditures, our study also highlights the different magnitudes of burden exerted by self-reported and undiagnosed NCDs. Whereas self-reported NCDs have significantly positive direct OOP and indirect burdens, the burden of undiagnosed hypertension and diabetes is virtually non-existent. This is likely because of lack of screening programs to detect these cases and poor or no treatment at early stages of disease. For example, undiagnosed hypertensive individuals had significantly lower outpatient utilization rates than non-hypertensive individuals. Since blood pressure measurement is common practice during outpatient visits, undiagnosed hypertensive individuals are likely to have not visited a healthcare provider and therefore to have gone undetected and untreated. Better screening programs will improve detection and long term health outcomes, yet they are also likely to increase overall treatment costs.

This study has several limitations worth noting. First, undiagnosed diabetes and heart problems were determined by self-assessed questions on symptomatic conditions rather than on actual clinical tests. The use of self-assessed questions may have led to significant under-reporting of prevalence such that the actual burden could be much greater than our forecasts indicate. Our method for identification of undiagnosed cases of diabetes and heart problems was based on the information available in IFLS; future studies should attempt to validate the accuracy of the algorithm in other data sets. There may also be significant under-reporting of healthcare utilization due to potential recall bias, although the extent of the bias is unknown. It is also important to note that our forecasting errors consider statistical uncertainty only; other forms of uncertainty, such as recall bias or measurement error, are not considered but may introduce additional error into the forecasts. Second, due to data limitations, the burden estimates do not include medical expenditures covered by insurance. About 29% of the study sample had some form of health insurance or benefit, ranging from social insurance to employer-provided clinics. Therefore it is possible that a sizeable portion of the direct costs of NCDs remains unaccounted for. Third, the indirect burden estimates did not include the value of premature mortality caused by NCDs, which is likely to be significant. For example, one study [32] reported that premature mortality attributable to CVDs in the United States is about 63% of direct costs. Estimating this burden for Indonesia should be an area of future research. Fourth, we applied linear forecasts to estimate what the burden of these NCDs would be in Indonesia in the absence of a significant health intervention. Linear time trends seemed to fit the data well but rapidly increasing rates of obesity and chronic disease in neighboring countries suggest this may be a very conservative assumption. Finally, our study is limited to those aged 40 and over, yet NCDs are increasingly being diagnosed among those in their 20′s and 30′s. For all of these reasons, the estimates presented here are likely to be a lower bound of future burden of these NCDs. Future studies will be needed to update these figures.

NCD incidence and an aging population play a major role in determining the burden of NCDs in Indonesia. The aging population is inevitable. Efforts to reduce the burden of NCDs will need to be multi-faceted and address both primary and secondary prevention, and treatment. It is, however, important to point out that the goal of investments in health is not necessarily to save money, but to improve the health of the population at a reasonable cost, such as below the commonly accepted value of 3 times per capita GDP per disability adjusted life year (DALY) as recommended by WHO [33] or the National Institute for Health and Clinical Excellence (NICE) threshold of £20,000 per Quality Adjusted Life Year (QALY) [34]. Within the contexts of NCDs, several promising public health and medical interventions that meet these thresholds exist. These include multi-drugs regimens for high-risk heart patients [35]–[36], target and self-monitored blood pressure control [37]–[38], screening for impaired glucose tolerance and intervening with either lifestyle or pharmacological interventions [39]–[40], bariatric surgery for severe obesity [41]–[42], and promoting a healthy lifestyle [43]–[45]. In addition to the proposed health reforms that Indonesia is already considering, expanding access to these interventions should be considered.

## Conclusion

In conclusion, this study demonstrates the significant burden of 4 primary NCDs on Indonesian households. In addition to the indirect burden, hypertension, diabetes, heart problems and stroke account for 8% of the nation's OOP healthcare expenditure, and due to rising disease prevalence and an aging population, this figure is expected to increase to 12% by 2020 without a significant health intervention.

## Supporting Information

Table S1 Linear time trends used for forecast gender- and age-specific prevalence rates.
(DOCX)
