# Supplementary material for: The Economic Burden of Self-Reported and Undiagnosed Cardiovascular Diseases and Diabetes on Indonesian Households
Source: PLoS One. 2014 Jun 10;9(6):e99572. doi: 10.1371/journal.pone.0099572 (PMC4051736; doi:10.1371/journal.pone.0099572)
Supplement: Table S1 — Linear time trends used for forecast gender- and age-specific prevalence rates. (DOCX) [file pone.0099572.s001.docx]

**Table S1.** Linear time trends used for forecast gender- and age-specific prevalence rates

| **Gender and age strata** | | **Hypertension (%)** | | **Diabetes (%)** | | **Heart problems (%)** | | **Stroke (%)** |
| --- | --- | --- | --- | --- | --- | --- | --- | --- |
|  |  | **SR [95% CI]** | **UD [95% CI]** | **SR [95% CI]** | **UD [95% CI]** | **SR [95% CI]** | **UD [95% CI]** | **SR [95% CI]** |
| Men aged 40 to 54 | |  |  |  |  |  |  |  |
|  | Baseline | 8.21 [8.03, 8.38] | 27.43 [27.27, 27.59] | 2.03 [1.98, 2.08] | 1.47 [1.46, 1.49] | 1.09 [1.05, 1.13] | 10.55 [10.49, 10.60] | 0.30 [0.28, 0.32] |
|  | Annual change | 0.11 [0.09, 0.13] | 0.23 [0.21, 0.25] | 0.04 [0.03, 0.04] | -0.01 [-0.01, 0.00] | 0.01 [0.01, 0.02] | -0.04 [-0.05, -0.04] | 0.00 [0.00, 0.01] |
| Women aged 40 to 54 | |  |  |  |  |  |  |  |
|  | Baseline | 17.43 [17.26, 17.60] | 26.11 [25.96, 26.26] | 2.14 [2.09, 2.19] | 1.70 [1.69, 1.72] | 2.00 [1.96, 2.04] | 11.65 [11.59, 11.70] | 0.71 [0.69, 0.73] |
|  | Annual change | 0.21 [0.19, 0.23] | 0.12 [0.10, 0.14] | 0.05 [0.04, 0.05] | 0.00 [-0.01, 0.00] | 0.00 [0.00, 0.01] | -0.02 [-0.03, -0.01] | 0.01 [0.01, 0.01] |
| Men aged 55 to 64 | |  |  |  |  |  |  |  |
|  | Baseline | 13.62 [13.36, 13.88] | 36.26 [36.03, 36.50] | 3.03 [2.95, 3.11] | 1.79 [1.76, 1.82] | 1.95 [1.88, 2.01] | 12.72 [12.63, 12.81] | 0.75 [0.72, 0.79] |
|  | Annual change | 0.20 [0.16, 0.23] | 0.23 [0.20, 0.26] | 0.08 [0.07, 0.09] | -0.01 [-0.01, 0.00] | 0.04 [0.04, 0.05] | -0.06 [-0.07, -0.05] | 0.01 [0.01, 0.02] |
| Women aged 55 to 64 | |  |  |  |  |  |  |  |
|  | Baseline | 23.75 [23.50, 23.99] | 35.52 [35.30, 35.74] | 2.79 [2.72, 2.87] | 1.88 [1.85, 1.91] | 3.49 [3.43, 3.55] | 15.46 [15.38, 15.54] | 1.27 [1.23, 1.30] |
|  | Annual change | 0.26 [0.23, 0.29] | 0.06 [0.03, 0.09] | 0.09 [0.08, 0.10] | 0.00 [0.00, 0.00] | 0.02 [0.01, 0.03] | -0.06 [-0.07, -0.05] | 0.03 [0.02, 0.03] |
| Men aged 65 to 74 | |  |  |  |  |  |  |  |
|  | Baseline | 19.48 [19.14, 19.83] | 41.42 [41.11, 41.74] | 3.43 [3.33, 3.54] | 2.25 [2.21, 2.29] | 2.83 [2.74, 2.91] | 14.47 [14.35, 14.58] | 1.71 [1.67, 1.75] |
|  | Annual change | 0.18 [0.14, 0.22] | 0.13 [0.09, 0.16] | 0.08 [0.06, 0.09] | 0.00 [0.00, 0.01] | 0.07 [0.06, 0.08] | -0.07 [-0.08, -0.05] | 0.04 [0.03, 0.05] |
| Women aged 65 to 74 | |  |  |  |  |  |  |  |
|  | Baseline | 26.66 [26.32, 26.99] | 46.68 [46.38, 46.98] | 2.99 [2.88, 3.09] | 1.44 [1.40, 1.48] | 2.96 [2.88, 3.04] | 16.48 [16.37, 16.59] | 0.65 [0.61, 0.69] |
|  | Annual change | 0.16 [0.12, 0.20] | -0.01 [-0.04, 0.03] | 0.07 [0.06, 0.08] | 0.00 [0.00, 0.00] | 0.01 [0.00, 0.02] | 0.05 [-0.07, -0.04] | 0.01 [0.01, 0.02] |
| Men aged 75 and above | |  |  |  |  |  |  |  |
|  | Baseline | 22.60 [22.05, 23.16] | 47.87 [47.37, 48.37] | 0.99 [0.82, 1.16] | 2.38 [2.32, 2.44] | 3.45 [3.31, 3.58] | 22.59 [22.40, 22.77] | 3.35 [3.28, 3.42] |
|  | Annual change | 0.09 [0.03, 0.16] | 0.06 [0.00, 0.12] | 0.02 [-0.01, 0.04] | 0.01 [0.00, 0.01] | 0.04 [0.03, 0.06] | -0.07 [-0.09, -0.05] | 0.05 [0.04, 0.06] |
| Women aged 75 and above | |  |  |  |  |  |  |  |
|  | Baseline | 26.76 [26.25, 27.27] | 49.85 [49.39, 50.31] | 1.20 [1.04, 1.36] | 1.58 [1.52, 1.63] | 3.21 [3.09, 3.34] | 20.90 [20.73, 21.07] | 1.04 [0.97, 1.10] |
|  | Annual change | 0.20 [0.14, 0.26] | -0.04 [-0.09, 0.02] | 0.04 [0.02, 0.06] | 0.01 [0.00, 0.02] | 0.01 [0.00, 0.03] | -0.10 [-0.12, -0.08] | 0.02 [0.02, 0.03] |

SR Self-reported; UD Undiagnosed

The baseline year is 1994
